# Alkaline sphingomyelinase (NPP7) impacts the homeostasis of intestinal T lymphocyte populations

**DOI:** 10.3389/fimmu.2022.1050625

**Published:** 2023-01-19

**Authors:** Manar Alyamani, Mohammad Kadivar, Jonas Erjefält, Bengt Johansson-Lindbom, Rui-Dong Duan, Åke Nilsson, Jan Marsal

**Affiliations:** ^1^ Immunology Section, Department of Experimental Medical Science, Lund University, Lund, Sweden; ^2^ Unit of Airway Inflammation, Department of Experimental Medical Science, Lund University, Lund, Sweden; ^3^ Department of Health Technology, Technical University of Denmark, Lyngby, Denmark; ^4^ Section of Medicine, Department of Clinical Sciences, Lund University, Lund, Sweden; ^5^ Department of Gastroenterology, Skane University Hospital, Lund/Malmö, Sweden

**Keywords:** alkaline sphingomyelinase, NPP7, inflammatory bowel disease, intestine, knockout, S1P, lymphocytes, T-cells

## Abstract

**Background and aim:**

Alkaline sphingomyelinase (NPP7) is expressed by intestinal epithelial cells and is crucial for the digestion of dietary sphingomyelin. NPP7 also inactivates proinflammatory mediators including platelet-activating factor and lysophosphatidylcholine. The aim of this study was to examine a potential role for NPP7 in the homeostasis of the intestinal immune system.

**Methods:**

We quantified the numbers of B-lymphocytes, plasma cells, T-lymphocytes including regulatory T-lymphocytes (T_regs_), natural killer cells, dendritic cells, macrophages, and neutrophils, in the small and large intestines, the mesenteric lymph nodes and the spleens of heterozygous and homozygous NPP7 knockout (KO) and wildtype (WT) mice. Tissues were examined by immunohistochemistry and stainings quantified using computerized image analysis.

**Results:**

The numbers of both small and large intestinal CD3ε^+^, CD4^+^, and CD8α^+^ T-lymphocytes were significantly higher in NPP7 KO compared to WT mice (with a dose-response relationship in the large intestine), whereas T_reg_ numbers were unchanged, and dendritic cell numbers reduced. In contrast, the numbers of CD3ε^+^ and CD4^+^ T-lymphocytes in mesenteric lymph nodes were significantly reduced in NPP7 KO mice, while no differences were observed in spleens. The numbers of B-lymphocytes, plasma cells, natural killer cells, macrophages, and neutrophils were similar between genotypes.

**Conclusion:**

NPP7 contributes to the regulation of dendritic cell and T-lymphocyte numbers in mesenteric lymph nodes and both the small and large intestines, thus playing a role in the homeostasis of gut immunity. Although it is likely that the downstream effects of NPP7 activity involve the sphingomyelin metabolites ceramide and spingosine-1-phosphate, the exact mechanisms behind this regulatory function of NPP7 need to be addressed in future studies.

## Introduction

Alkaline sphingomyelinase, also named nucleotide pyrophosphatase phosphodiesterase 7 (NPP7) (1), is an ectoenzyme that was discovered and characterized by our group (2, 3), is expressed selectively by the intestinal and in humans biliary epithelia, and is important for the digestion of dietary sphingomyelin (SM) (4). While the colon displays some NPP7 activity, the highest activity is seen in the small intestine (4). The enzyme, which is protease-resistant, can be shed from the epithelia where it is expressed, and enzymatic activity can readily be detected in both bile and feces (5).

In addition to SM, other substrates for NPP7 are platelet activating factor (PAF) and lysophosphatidylcholine (lyso-PC) which both are proinflammatory mediators (1). NPP7 has the ability to cleave the phosphocholine headgroup off from these phospholipids, producing various lipid metabolites. The enzyme thus has several branches of downstream metabolic pathways with the generation of a number of lipid messengers that have different biologic effects. Ceramide and sphingosine, which are produced when NPP7 digests either exogenous or endogenous SM, are lipid mediators that inhibit cell proliferation, induce apoptosis, and may counteract carcinogenesis (6). A downstream metabolite of ceramide and sphingosine is sphingosine-1-phosphate (S1P) which has been implicated in angiogenesis, innate and adaptive immunity, lymphocyte trafficking, and the pathophysiology of inflammatory bowel disease through contributing to the activation of the IL-6/STAT3 and NFκB pathways, as well as the pathogenesis of colon cancer (7). S1P has effects both locally in the intestinal mucosa as well as in lymph nodes where it directs the egress of lymphocytes from the lymph nodes to efferent lymphatic vessels from where the cells are transported to the blood circulation (7).

By hydrolysis, NPP7 can inactivate PAF which has proinflammatory properties including MAP kinase activation, chemotaxis, and cytokine release, and has been implicated in ulcerative colitis (8). By converting lyso-PC to monoacylglycerol, NPP7 activity leads to decreased formation of lysophosphatidic acid (LPA) which has several proinflammatory effects including activation of Ras, Rac, and PI3 kinase (4, 9). Thus, NPP7 activity is thought to have both anti-inflammatory and anti-carcinogenic effects (1). Indeed, decreased NPP7 activity levels have been found in ulcerative colitis, colonic carcinoma, sporadic adenomas, and familial adenomatous polyposis (FAP) (1). Furthermore, in a rat dextran sulphate sodium (DSS) colitis model, rectal instillation of recombinant NPP7 was shown to alleviate colitis activity (10), and in a murine DSS colitis model NPP7 knockout (KO) mice were shown to have more severe colitis activity with increased levels of PAF, LPA, and autotaxin (8). Finally, dietary SM has been shown to inhibit carcinogen-induced colon cancer in an animal model (11), and colitis-associated colon cancer was enhanced in NPP7 KO mice (12).

In contrast to most other tissues in the body, the gut is characterized by an ever present physiological low-grade degree of immunological activity, maintaining a balance between immunity to pathogens and neoplastic epithelial cells on the one hand, and immune tolerance to innocuous antigens from food and commensal bacteria on the other (13). A large number of various immune cell populations are normally present and cooperate in a complex network in the gut mucosa to keep this balance, including CD4^+^ and CD8^+^ T-cells, regulatory T-cells (T_reg_), B-cells, natural killer (NK) cells, innate lymphoid cells (ILC), plasma cells, macrophages, and dendritic cells (DC) (13). Naïve lymphocytes are activated and proliferate in draining mesenteric lymph nodes or Peyer’s patches, and home subsequently to the gut mucosa (14). Understanding how this network is regulated is of highest importance, since numerous severe diseases, including inflammatory bowel disease and colitis-associated colorectal cancer, emerge when there is a dysregulation of the system (13).

The effects of NPP7 deficiency on the gut-related immune system under homeostatic conditions have not been studied. Therefore, the aim of this study was to characterize the immunological phenotype of NPP7 KO mice, by examining major immune cell populations in NPP7-related immunological compartments using computer-assisted quantitative image analysis of immune-stained tissue sections from homozygous and heterozygous NPP7 deficient mice, and wildtype mice. The results showed significant differences primarily in T-lymphocyte populations in specific anatomical compartments, suggesting a role for NPP7 in the homeostasis of intestinal immunity.

## Materials and methods

### Animals

NPP7 deficient mice were originally generated by members of our group (Duan and his coworkers) at our university using the Cre-LoxP system, as previously described (5). C57BL/6 NPP7 heterozygous (HT) mice were used for breeding, and littermates were used for experiments. The genotypes of the littermates were defined by PCR as previously described (5). PCR results, protein expression, and NPP7 enzyme activity has previously been confirmed to fully correlate (5). All mice were bred and housed under specific pathogen-free conditions in isolated ventilated cages at the Biomedical Centre (BMC), Lund University, Lund, Sweden. Mice were weaned at 3 weeks of age and fed commercial standard pellets with free access to water. Tissues were collected from 16 NPP7^+/+^ wildtype (WT; female [F]/male [M] ratio 6/10), 27 NPP7^+/−^ HT (F/M 15/12), and 23 NPP7^−/−^ KO (F/M 9/14) mice, at 5 weeks of age. Mice were euthanized by cervical dislocation under complete isoflurane inhalation anesthesia and sedation. All procedures were performed with the consent from the regional animal research ethics committee (M57-08, M177-10) and in accordance with Swedish animal protection laws.

### Tissue collection and preparation

The middle 4 cm of the small and large intestine, respectively, the mesenteric lymph nodes (MLN), and the spleen were collected from each mouse. Tissues were fixed in phosphate buffered saline (PBS) with 4% paraformaldehyde for 20 hours at room temperature (RT), dehydrated through a series of immersions in graded ethanol, and embedded in paraffin. The tissue blocks were sectioned (thickness 4 μm) using a microtome (Thermo Fisher Scientific) and slides placed onto a waterbath (40°C) for 2 minutes to reduce wrinkles. Sections were transferred to positively charged slides (Dako FLEX IHC Microscope Slides, Dako), and left to dry (24 hours at RT). Slides were heated in an oven at 60°C for 30 minutes, followed by deparaffinization with xylene and then rehydration. Antigen retrieval was performed by incubations in either high or low pH heat-induced epitope retrieval solution (HIER; DAKO-DM829, Dako) in a HIER apparatus (PT-link 200, Dako). Slides were allowed to cool at room temperature for 30 minutes, followed by a wash in PBS.

### Immunostaining of tissues

Tissue sections were encircled by a hydrophobic barrier using a PAP pen. Sections were incubated with hydrogen peroxide for 10 minutes at RT, followed by a 20-minute incubation with 2.5% normal horse serum (Vector Laboratories), and either 2.5% normal goat serum or Rodent Block M (BioCare) depending on whether the secondary antibody host was goat or rat, respectively. Sections were incubated with a primary antibody either 1 hour at RT or overnight at 4°C. Isotype-matched antibodies were used as negative controls. Primary antibodies (clone) detecting the following antigens were used: CD3ε (CD3-12, Abcam; polycloncal, Thermo Fisher Scientific); CD4 (50134-R001, SinoBiological); CD8α (D4W2Z, Cell Signaling); FoxP3 (D6O8R, Cell Signaling); CD19 (D4V4B, Cell Signaling); B220 (RA3-6B2, Thermo Fisher Scientific); CD138 (mSDC1, R&D); IgA (RM220, Novus Biologics); CD11c (D1V9Y, Cell Signaling); CD163 (EPR19518, Abcam); F4/80 (D2S9R, Cell Signaling); Zap70 (99F2, Cell Signaling); myeloperoxidase (PA5-16672, Thermo Fisher Scientific). For some stainings, antibodies to CD19 and B220, and CD163 and F4/80, respectively, were applied as cocktails. After incubation with the primary antibody, sections were washed with PBS wash buffer, and immunoreactivity was visualized using the secondary antibody ImmPRESS® peroxidase (HRP) polymer detection system according to the manufacturer’s instructions (Vector Laboratories) in combination with chromogenic substrates (DAB [Dako] for single staining, and Vina Green [BioCare] in addition for double staining). After chromogen development, slides were counterstained with hematoxylin, and coverslipped.

### Computerized quantitative image analysis

Immunohistochemically stained tissue sections were digitized using a slide-scanning robot (ScanScope, Aperio Technologies). High-resolution digital images of the entire section areas were generated for all mice, organs, and tissue sections, and subjected to computerized quantitative analysis. Immunoreactivity-positive areas and negative tissue areas were color-coded and processed using the software Visiomorph™ (Visiopharm) as shown in **Supplementary Figure E**. Relevant tissue areas to be analyzed were designated Region of Interest (ROI) for intestinal and secondary lymphoid tissues, respectively, as shown in **Supplementary Figures F, G**. Immunoreactivity-positive signals were quantified using Visiomorph™. Positive areas were normalized to the ROI analyzed, and data are presented as positive tissue fraction (positivity, %). The positivity directly reflects the number of cells, and these terms are thus used interchangeably.

Certain celltypes were defined by being positive for some specific cellular markers while being negative for others. This was made possible digitally in the computerized analysis by quenching the areas positive for markers denoting irrelevant cells leaving only the celltype of interest to be quantified. Quenched areas were included in the denominator area-value denoting the entire tissue section.

### Statistical analysis

Data are presented as individual data points and as group mean values with standard deviations unless otherwise stated. Each individual data point is the mean value of two separate analyses of the same mouse and tissue. Comparisons between groups were tested for statistical significance using a one-way ANOVA with Tukey’s multiple comparison test. Data sets were examined regarding distribution characteristics using the D’Agostino-Pearson normality test before applying a parametric test. *P*<0.05 was considered statistically significant. Statistical analyses were performed using GraphPad Prism version 9.4.1 for MacOS (GraphPad Software).

## Results

### NPP7 deficient mice have increased numbers of T-lymphocytes in the small intestinal mucosa, whereas dendritic cell numbers are decreased

**Figure 1:**
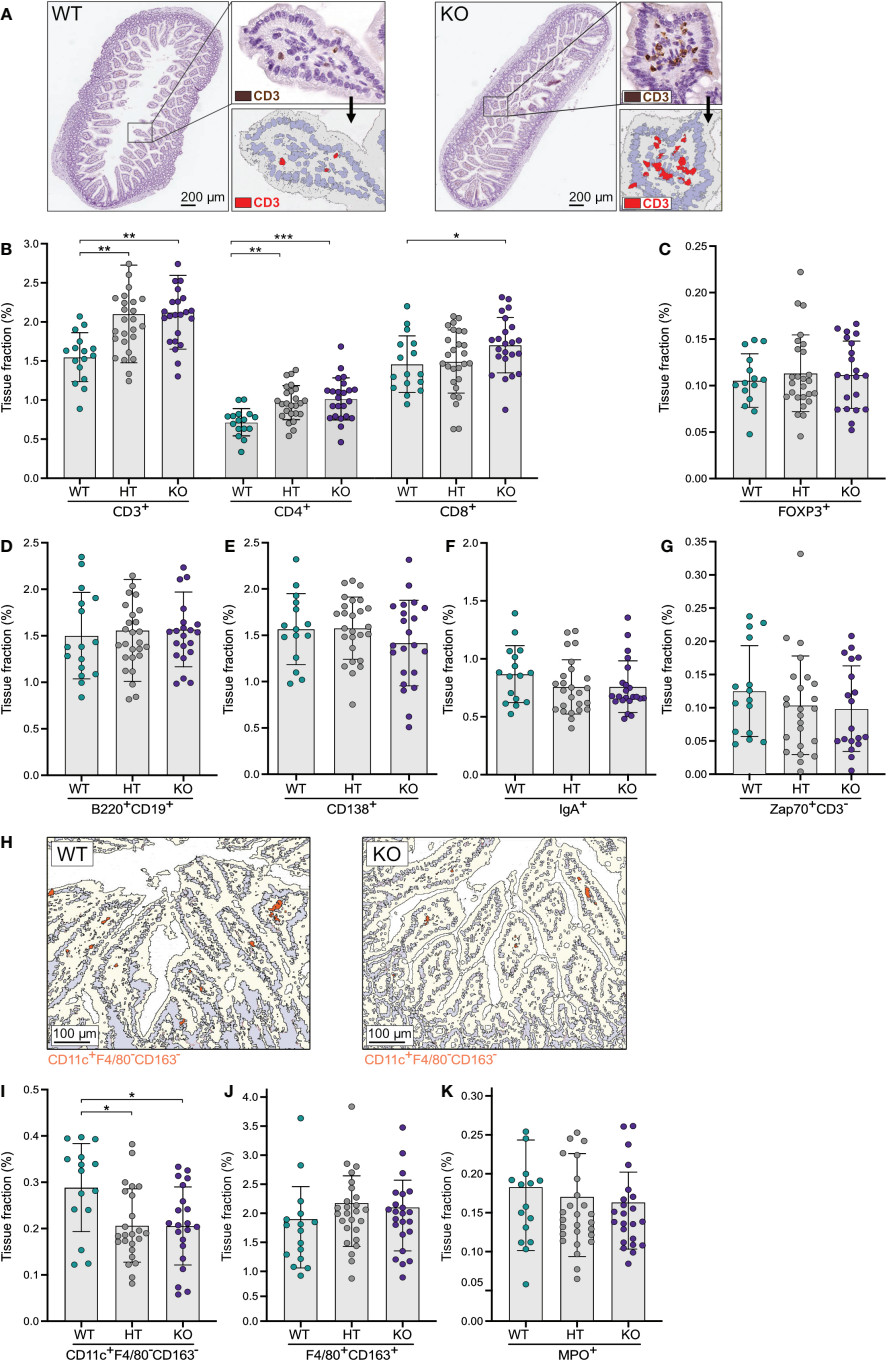
Quantitative analyses of immune cells in the small intestinal mucosa showed an increase in T-lymphocytes in NPP7 KO mice. Small intestines from NPP7 HT and KO mice, and WT mice, were stained for various immune cell populations by immunohistochemistry. Computerized image analysis was used to quantify positivity (stained fraction of tissue area) reflecting cell numbers. **(A)** Representative bright-field images of small intestines from WT mouse (left) and NPP7 KO mouse (right), out of 32 and 46 images, respectively, illustrating the staining for CD3ε^+^ T-lymphocytes in brown (DAB chromogen) and the quantitative image analysis for CD3ε^+^ T-lymphocytes shown in red; **(B)** CD3ε^+^, CD4^+^ and CD8α^+^ T-lymphocytes; **(C)** Foxp3^+^ T_regs_; **(D)** B-lymphocytes; **(E)** CD138^+^ plasma cells; **(F)** IgA^+^ cells; **(G)** Zap70^+^CD3ε^−^ NK cells; **(H)** Representative images of small intestine from WT mouse (left) and NPP7 KO mouse (right), out of 32 and 46 images, respectively, illustrating the quantitative image analysis for CD11c^+^F4/80^−^CD163^−^ DCs shown in orange; **(I)** CD11c^+^F4/80^−^CD163^−^ DCs; **(J)** Macrophages; and **(K)** MPO^+^ neutrophils. Each dot represents an individual mouse and is the mean of two staining experiments. Columns show the group mean ± SD. *P<0.05, **P<0.001, ***P<0.001.

To assess a potential role for NPP7 in the homeostasis of gut T-lymphocytes, small intestinal tissues were stained for CD3ε and computerized image analysis was applied to quantify the expression (**Figure 1A**). The positive tissue fractions, which reflect the numbers of cells, were compared between WT, HT, and KO mice. The number of small intestinal CD3ε^+^ cells was approximately 36% higher in KO and HT mice compared to WT mice (**Figure 1B**). Analysis of T-lymphocyte subsets, staining for CD4 and CD8α, showed similar differences for both subsets, but with a somewhat less clear impact on the CD8α^+^ T-lymphocyte population (**Figure 1B**). By contrast, regulatory T-lymphocytes (T_regs_) defined as FoxP3^+^ cells did not show differences between the groups (**Figure 1C**).

Investigating the numbers of B-lymphocytes (stained by a cocktail of B220 and CD19), CD138^+^ plasma cells, IgA^+^ cells, and Zap70^+^CD3ε^−^ natural killer (NK) cells in the small intestinal mucosa, we found no significant differences between the groups, although the CD138^+^, IgA^+^, and Zap70^+^CD3ε^−^ cells were numerically fewer in the KO mice (**Figures 1D–G**).

Examining myeloid cell populations in the small intestine, we found a significant decrease in the number of CD11c^+^F4/80^−^CD163^−^ dendritic cells (DCs) in NPP7 KO and HT mice (**Figure 1H**), but macrophages (stained by a cocktail of F4/80 and CD163 antibodies) and neutrophils (MPO^+^ cells) did not show significant differences between the groups (**Figures 1I–K**).

### NPP7 deficiency has similar effects on T-lymphocyte populations in the large intestine as in the small intestine

**Figure 2:**
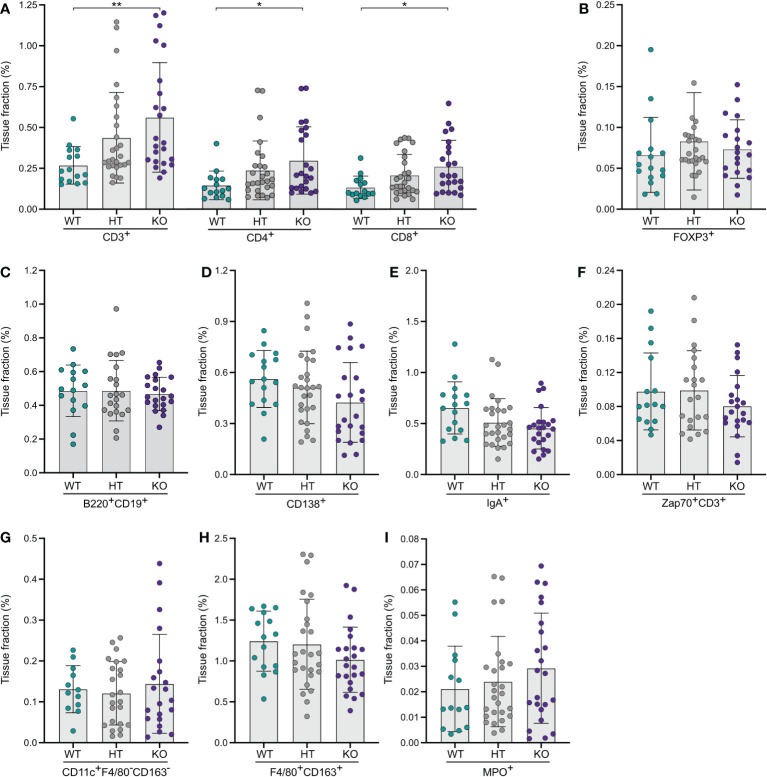
Quantitative analyses of immune cells in the large intestinal mucosa of NPP7 KO mice showed a doubling in T-lymphocyte numbers. Large intestines from NPP7 HT and KO mice, and WT mice, were stained for various immune cell populations by immunohistochemistry. Computerized image analysis was used to quantify positivity (stained fraction of tissue area) reflecting cell numbers. **(A)** CD3ε^+^, CD4^+^ and CD8α^+^ T-lymphocytes; **(B)** Foxp3^+^ T_regs_; **(C)** B-lymphocytes; **(D)** CD138^+^ plasma cells; **(E)** IgA^+^ cells; **(F)** Zap70^+^CD3ε^−^ NK cells; **(G)** CD11c^+^F4/80^−^CD163^−^ DCs; **(H)** Macrophages; and **(I)** MPO^+^ neutrophils. Each dot represents an individual mouse and is the mean of two staining experiments. Columns show the group mean ± SD. *P<0.05, **P<0.001.

Computerized quantification of the expression of T-lymphocyte markers showed that KO mice had more than twice as many (209%) CD3ε^+^ T-lymphocytes in the large intestinal mucosa compared to WT mice (**Figure 2A**). Analyses of CD4^+^ and CD8α^+^ T-lymphocyte subsets showed similar findings (**Figure 2A**). There was a distinct dose-response relationship observed with regards to no NPP7 deficiency, and heterozygous and homozygous NPP7 deficiency, for CD3ε^+^, CD4^+^, and CD8α^+^ T-lymphocytes, respectively (**Figure 2A**). By contrast, there were no differences in the number of FoxP3^+^ T_regs_ between the groups (**Figure 2B**).

Quantifications of B-lymphocytes, CD138^+^ plasma cells, IgA^+^ cells, and Zap70^+^CD3ε^−^ NK cells in the large intestinal mucosa showed no significant differences between the groups, although the CD138^+^, IgA^+^, and Zap70^+^CD3ε^−^ cells were numerically fewer in the KO mice (**Figures 2C–F**). Similarly, the myeloid cell populations in the large intestine including DCs, macrophages, and neutrophils did not display significant differences between the groups (**Figures 2G–I**).

### Mesenteric lymph nodes of NPP7 deficient mice display reduced numbers of T-lymphocytes and dendritic cells

**Figure 3:**
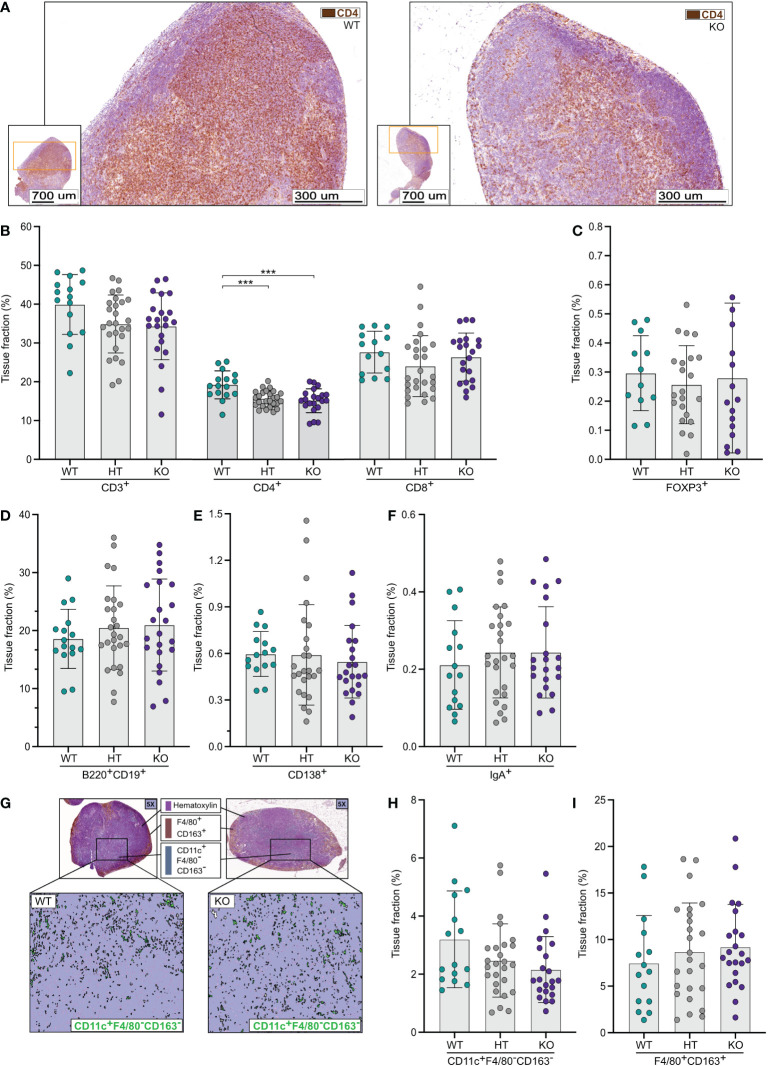
Quantitative analyses of immune cells in mesenteric lymph nodes (MLNs) showed a reduction in CD4^+^ T-lymphocytes in NPP7 KO mice. MLNs from NPP7 HT and KO mice, and WT mice, were stained for various immune cell populations by immunohistochemistry. Computerized image analysis was used to quantify positivity (stained fraction of tissue area) reflecting cell numbers. **(A)** Representative images of MLNs from WT mouse (left) and NPP7 KO mouse (right), out of 32 and 46 images, respectively, illustrating the staining of CD3ε^+^ T-lymphocytes in brown (DAB chromogen); **(B)** CD3ε^+^, CD4^+^ and CD8α^+^ T-lymphocytes; **(C)** Foxp3^+^ T_regs_; **(D)** B-lymphocytes; **(E)** CD138^+^ plasma cells **(F)** IgA^+^ cells; **(G)** Representative images of MLNs from WT mouse (left) and NPP7 KO mouse (right), out of 32 and 46 images, respectively, illustrating the staining of macrophages in brown (DAB chromogen) and DCs in green (Vina green chromogen) [upper panels], and quantitative image analysis of CD11c^+^F4/80^−^CD163^−^ DCs shown in green with the macrophage signal quenched [lower panels]; **(H)** CD11c^+^F4/80^−^CD163^−^ DCs; and **(I)** Macrophages. Each dot represents an individual mouse and is the mean of two staining experiments. Columns show the group mean ± SD, ***P<0.001.

In contrast to the results from the small and large intestines, quantitative image analyses of mesenteric lymph nodes (MLNs) (**Figure 3A**) showed a numerical reduction of CD3ε^+^ T-lymphocytes in NPP7 KO and HT mice compared to WT mice but the differences were not statistically significant (**Figure 3B**). Analysis of CD4^+^ T-lymphocytes showed a similar pattern as for CD3ε^+^ T-lymphocytes but with statistically significant differences (**Figure 3B**), whereas CD8α^+^ T-lymphocyte levels were similar between the groups (**Figure 3B**). The latter was true for the FoxP3^+^ T_regs_ as well (**Figure 3C**).

Analyses of B-lymphocytes, CD138^+^ plasma cells, and IgA^+^ cells (**Figures 3D–F**), and Zap70^+^CD3ε^−^ NK cells (**Supplementary Figure A**) in MLNs showed similar numbers of cells comparing WT, HT, and KO mice.

Quantifications of CD11c^+^F4/80^−^CD163^−^ DCs in MLNs (**Figure 3G**) showed numerically lower numbers in NPP7 HT and KO mice compared to WT mice, but the differences were not statistically significant (**Figure 3H**). The numbers of macrophages (**Figure 3I**) and neutrophils (**Supplementary Figure B**) in MLNs were similar between the groups.

### NPP7 deficiency does not affect lymphoid or myeloid cell populations in the spleen

**Figure 4:**
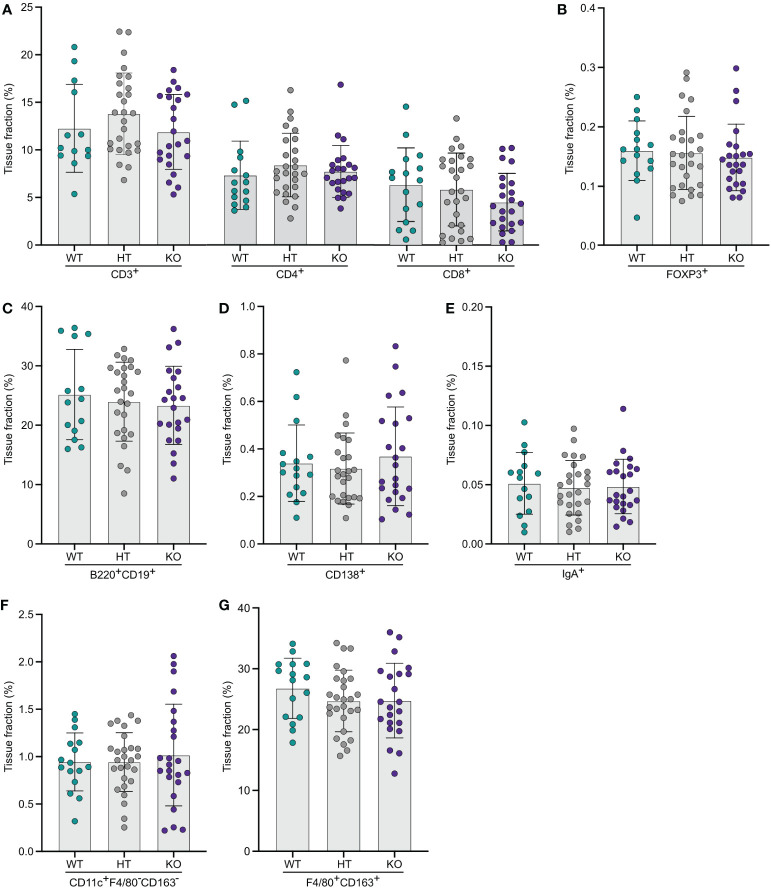
Quantitative analyses of immune cells in spleens did not show any significant differences comparing WT and NPP7 deficient mice. Spleens from NPP7 HT and KO mice, and WT mice, were stained for various immune cell populations by immunohistochemistry. Computerized image analysis was used to quantify positivity (stained fraction of tissue area) reflecting cell numbers. **(A)** CD3ε^+^, CD4^+^ and CD8α^+^ T-lymphocytes; **(B)** Foxp3^+^ T_regs_; **(C)** B-lymphocytes; **(D)** CD138^+^ plasma cells; **(E)** IgA^+^ cells; **(F)** CD11c^+^F4/80^−^CD163^−^ DCs; and **(G)** Macrophages. Each dot represents an individual mouse and is the mean of two staining experiments. Columns show the group mean ± SD.

The same type of computerized quantitative image analyses as for the other organs were performed for spleens from WT, and NPP7 HT and KO mice. No significant differences between the groups were observed for CD3ε^+^, CD4^+^, or CD8α^+^ T-lymphocytes, FoxP3^+^ T_regs_, B-lymphocytes, CD138^+^ plasma cells, IgA^+^ cells, CD11c^+^F4/80^−^CD163^−^ DCs, macrophages (**Figures 4A–G**), Zap70^+^CD3ε^−^ NK cells or neutrophils (**Supplementary Figures C, D**).

## Discussion

In the current study, we quantified major immune cell populations of the small and large intestines, the MLNs, and the spleens of NPP7 HT and KO mice, and WT mice, by means of quantitative image analysis, with the aim to investigate a potential role for NPP7 in the homeostasis of the gut immune system, which in turn is of central importance in numerous diseases including inflammatory bowel disease and colorectal cancer. Interestingly, the numbers of T-lymphocytes were increased by 36% in the small intestinal mucosa and by 109% in the colonic mucosa of NPP7 KO mice compared to WT mice, whereas T-lymphocyte numbers were similar or decreased in the MLNs of NPP7 KO mice. Numbers of DCs in NPP7 KO mice were significantly decreased in the small intestine, but did not show significant changes in the MLNs. The other immune cell populations examined, including T_regs_, were similar in numbers between genotypes. These data demonstrate that NPP7 plays an important role in regulating the homeostasis of effector T-lymphocytes of the gut mucosa.

Several studies have shown an association between reduced NPP7 levels and colonic inflammation as well as adenocarcinoma. Patients with ulcerative colitis had decreased NPP7 levels in biopsies compared to controls (15) and low levels of NPP7 have also been found in colon cancer as well as fecal samples from patients with colon cancer (16, 17). Conversely, when recombinant NPP7 was given rectally, colitis was alleviated in a rat DSS-colitis model (10), and in calves it was shown that an upregulation of NPP7 is part of an immunosuppressive response to asymptomatic chronic enteric colonization with enterohemorrhagic *Escherichia coli* (18). NPP7 thus plays a role in counteracting intestinal inflammation and carcinogenesis, but the exact mechanisms behind these functions are not fully elucidated.

To link our findings to currently known functions of NPP7, several potential mechanisms may be suggested. NPP7 is known to hydrolyze and inactivate PAF, a paracrine messenger that stimulates leukocyte as well as lymphocyte migration (19). Furthermore, NPP7 hydrolyzes lyso-PC to monoglyceride and phosphocholine whereas autotaxin (NPP2), an ectoenzyme belonging to the same family as NPP7, hydrolyzes lyso-PC to generate LPA, a paracrine messenger that stimulates cell migration and proliferation (20). During induction of colitis with DSS, NPP7 KO mice exhibited an earlier rise in PAF in the colonic mucosa compared to WT mice (8). Mucosal LPA and autotaxin also increased more in NPP7 KO mice, but with a slower time course than PAF (8).

Chen et al. showed that NPP7 KO mice had a significant reduction of ceramide levels in the small intestine but not the colon (12), which is in agreement with NPP7 expression levels being higher in the small intestine as compared with the colon (4). Our data on the other hand showed a stronger effect of NPP7 deficiency on the T-lymphocyte numbers in the colon as compared with the small intestine.

Increased S1P-levels have been observed in various inflammatory diseases, mediating recruitment of immune cells and enhancing inflammation, but immune-regulatory functions have also been assigned to S1P (21–23). The regulation of S1P-levels in tissues is complex, involves several separate metabolic pathways, and can be adjusted at either steps of synthesis or degradation (23). Absorbed sphingosine, generated from the sequential action of NPP7 and mucosal ceramidase, is converted in the mucosa to S1P, most of which is further metabolized to palmitaldehyde and ultimately to palmitic acid and incorporated into chylomicron triglyceride (24). Nevertheless, some of the generated S1P may exert effects in the mucosal compartment *via* both paracrine receptor-mediated and direct intracellular signalling, and thereby regulate lymphocyte recruitment (25, 26). S1P may act directly on immune cells, but may also regulate T-cell recruitment by affecting mucosal endothelial cells, which also express S1P-receptors (23, 27). Although S1P has local mucosal effects, S1P has primarily been studied with regards to its effects in lymph nodes, where S1P mediates the egress of lymphocytes, and when S1P-receptors are pharmacologically downregulated, lymphocytes are prevented from leaving the lymph node leading to a systemic decrease in lymphocyte numbers (22, 28, 29). Intracellular enzymes including other sphingomyelinases, i.e. sphingomyelin phosphodiesterases (SMPD) 1-5, and sphingosine kinases (SPHK) 1-2 are also important for the regulation of S1P-levels and could potentially change their activity when NPP7 is deleted (30).

Interestingly, Chen et al. found an increase in S1P-levels in the small intestine of NPP7 KO mice, and the increase was even more pronounced in the colon (31). It is thus conceivable that the altered T-lymphocyte numbers in the intestinal mucosa and in the draining mesenteric lymph nodes that we observed in NPP7 KO mice are directly or indirectly related to changes in S1P-levels (32, 33). In contrast, NPP7 does not seem to have major systemic effects in terms of cell numbers, given that splenic immune cell populations were quantitatively unaltered in NPP7 KO mice as compared with WT mice. Of note, our data showed a decrease in DC-numbers in both the intestine and the MLNs, which is an additional possibility as to how T-lymphocyte numbers may have been affected. Intestinal DCs are known to have important immune regulatory functions which potentially could include limiting T-lymphocyte numbers with opposite effects if DCs are absent due to NPP7 deficiency (34). Additional possible explanations to altered T-lymphocyte and DC numbers as a result of NPP7 deficiency include effects on proliferation, apoptosis, and retention in tissue.

Other candidate mechanisms that may contribute to explaining our results can be found in a recent report by Zhu et al. where a comparison of the intestinal transcriptome in WT and NPP7 KO mice was presented (35). Ninety-seven genes were differentially expressed, including genes that are linked to metabolism and absorption as well as immune regulation. Two factors that showed statistically significant differences in expression levels, and were highlighted by the authors, were SPP1 (osteopontin) and H2-AB1 (an MHC-II molecule). Interestingly, osteopontin, which is important for bone remodeling, is also a potent proinflammatory mediator that has been strongly implicated in the recruitment of T-cells to the intestinal mucosa and shown to have a central role in intestinal inflammation and regeneration (36–38). Intestinal H2-AB1 expression may be accounted for by professional antigen-presenting cells, but also by intestinal epithelial cells, innate lymphoid cells (ILC) and the recently described Thetis cells, and has been shown to be affected by dietary modifications (39–41). The data regarding the effects of epithelial and mucosal H2-AB1 expression on T-cell numbers and intestinal inflammation are diverging, and studies have shown associations with both an increase and decrease (41–43).

Taken together, NPP7 KO mice are more susceptible to induction of intestinal inflammation and proinflammatory as well as protective factors (e.g. ceramide, S1P, osteopontin and H2-AB1) are differentially expressed in NPP7 KO mice already before a colitis-inducing challenge. To dissect the exact sequences of events that link NPP7 activity to intestinal T-cell numbers and inflammatory activity will require elaborate experiments including time course studies on immune cell populations, gene and protein expression, and levels of lipid messengers and of various enzymes involved in sphingosine metabolism, after inflammatory challenge.

This study has several limitations. First, mice were only analyzed at a single time-point. It would have been interesting to perform the analyses at several time-points to reveal potential dynamics in the changes, including a possible effect of the time-point for weaning of the mice, at which the gut goes from being exposed to vast amounts of SM through milk, to lesser exposure. Second, the study would have benefited from performing flow cytometry analyses on immune cell populations from the intestines and MLNs in parallel, to investigate in greater detail which subpopulations were primarily affected. Third, it would have been interesting to analyze the intestinal epithelial compartment separately from the lamina propria to investigate whether intraepithelial T-lymphocyte (IEL) populations were more or less severely affected by NPP7 deficiency compared to the lamina propria populations, both because of the microanatomical vicinity to the enzymatic activity of NPP7 and because the IELs comprise unconventional subpopulations that could be differentially affected compared to conventional T-lymphocytes. Of note, our data suggested a similarly strong or even stronger effect on CD4^+^ T-lymphocytes as compared to CD8α^+^ T-lymphocyte which could be interpreted as conventional T-lymphocytes being primarily affected. Finally, it would have been interesting to quantify some of the known downstream metabolites of NPP7 activity in the various anatomical locations examined, to investigate whether there was a correlation between the effect and metabolite levels proposing a possible mechanistic link.

The current study has several strengths. First, the number of mice analyzed is high compared to similar studies that aim to investigate an immunologic phenotype of a KO mouse. Second, we analyzed both heterozygous and homozygous mice, which showed a dose-response relationship between the genotype and the magnitude of effect. Third, applying quantitative image analysis to quantify the number of immune cells generated data showing absolute quantities. Finally, analysis of both the small and large intestines together with the locally draining MLNs (which provide the intestines with newly activated T-lymphocytes) in combination with the spleen representing the systemic immunological state being less dependent on the events in the gut where NPP7 is expressed, together comprised a robust study design.

In conclusion, this study strongly suggests that NPP7 is instrumental in regulating the numbers of T-lymphocytes in both the small and large intestines, and thus potentially important for keeping the homeostasis of the gut immune system and for downregulating proinflammatory immune responses.

## Data availability statement

The original contributions presented in the study are included in the article/**Supplementary Material**. Further inquiries can be directed to the corresponding author.

## Ethics statement

The animal study was reviewed and approved by the regional animal research ethics committee (M57-08, M177-10) and in accordance with Swedish animal protection laws.

## Author contributions

MA, MK, R-DD, ÅN, JM contributed to conception and design of the study. JE, BJ-L, R-DD, JM contributed reagents/analytic tools. MA, MK, BJ-L, JM worked with the animals, processed samples and performed experiments. MA, MK, JE, JM carried out the data management and statistical analysis. MA, MK, JE, BJ-L, R-DD, ÅN, JM analyzed and interpreted data. MA, JM wrote the first draft of the manuscript. JE, JM supervised the work. All authors contributed to the article and approved the submitted version.

## Supplementary material

Supplementary Figure Quantitative analyses of NK cells and neutrophils in MLNs and spleens did not show differences comparing WT and NPP7 deficient mice. MLNs and spleens were stained by immunohistochemistry. Computerized image analysis was used to quantify positivity (stained fraction of tissue area) reflecting cell numbers. **(A)** Zap70^+^CD3ε^−^ NK cells in MLNs; **(B)** MPO^+^ neutrophils in MLNs; **(C)** Zap70^+^CD3ε^−^ NK cells in spleens; **(D)** MPO^+^ neutrophils in spleens; **(E)** Micrographs of small intestinal tissue illustrating pre-analysis image processing workflow for training the software to classify positive and negative signals, respectively; **(E1)** Target cell stained with brown chromogen [DAB chromogen] and nuclei identified through counter staining with hematoxylin; **(E2)** Initial manual classification of signals (dark brown classified as target cell, labeled with green; light grey classified as negative, labeled with light blue; purple classified as nuclei, labeled with purple); **(E3)** Final automated classification of signals by software (both lighter brown and dark brown classified as target cell, labeled with green; all grey area classified as negative, labeled with light blue; all purple areas classified as nuclei, labeled with purple); **(F)** Image demonstrating region of interest (ROI), i.e. the mucosal compartment, in an intestinal section with the muscularis layer and lumen omitted (ROI in solid grey); **(G)** Image demonstrating region of interest (ROI) in MLN section (ROI defined by dotted red line). Each dot in the column graphs represents an individual mouse and is the mean of two staining experiments. Columns show the group mean ± SD. *P<0.05, **P<0.001, ***P<0.001.
