# Supplementary figures and images for: Alkaline sphingomyelinase (NPP7) impacts the homeostasis of intestinal T lymphocyte populations
Source: Front Immunol. 2023 Jan 19;13:1050625. doi: 10.3389/fimmu.2022.1050625 (PMC9894718; doi:10.3389/fimmu.2022.1050625)

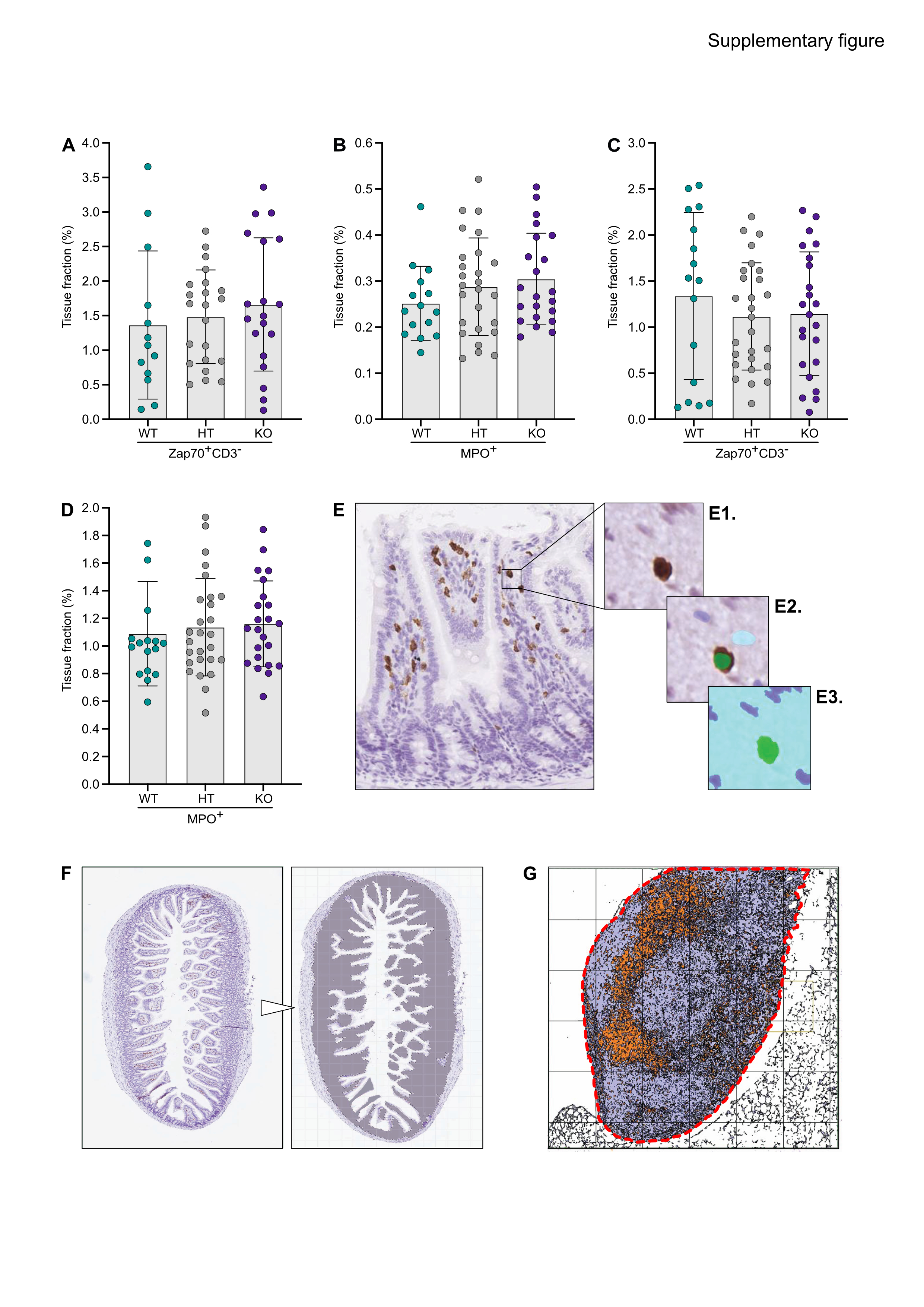

Supplement: Supplementary Figure — Quantitative analyses of NK cells and neutrophils in MLNs and spleens did not show differences comparing WT and NPP7 deficient mice. MLNs and spleens were stained by immunohistochemistry. Computerized image analysis was used to quantify positivity (stained fraction of tissue area) reflecting cell numbers. (A) Zap70+CD3ε− NK cells in MLNs; (B) MPO+ neutrophils in MLNs; (C) Zap70+CD3ε− NK cells in spleens; (D) MPO+ neutrophils in spleens; (E) Micrographs of small intestinal tissue illustrating pre-analysis image processing workflow for training the software to classify positive and negative signals, respectively; (E1) Target cell stained with brown chromogen [DAB chromogen] and nuclei identified through counter staining with hematoxylin; (E2) Initial manual classification of signals (dark brown classified as target cell, labeled with green; light grey classified as negative, labeled with light blue; purple classified as nuclei, labeled with purple); (E3) Final automated classification of signals by software (both lighter brown and dark brown classified as target cell, labeled with green; all grey area classified as negative, labeled with light blue; all purple areas classified as nuclei, labeled with purple); (F) Image demonstrating region of interest (ROI), i.e. the mucosal compartment, in an intestinal section with the muscularis layer and lumen omitted (ROI in solid grey); (G) Image demonstrating region of interest (ROI) in MLN section (ROI defined by dotted red line). Each dot in the column graphs represents an individual mouse and is the mean of two staining experiments. Columns show the group mean ± SD. *P<0.05, **P<0.001, ***P<0.001. [file Image_1.jpeg]
